# A CMOS PSR Enhancer with 87.3 mV PVT-Insensitive Dropout Voltage for Sensor Circuits

**DOI:** 10.3390/s21237856

**Published:** 2021-11-25

**Authors:** Jianyu Zhang, Pak Kwong Chan

**Affiliations:** School of Electrical and Electronic Engineering, Nanyang Technological University, Singapore 639798, Singapore; zh0028yu@e.ntu.edu.sg

**Keywords:** PSR enhancer, regulator, dropout voltage, temperature compensation, current reference, voltage reference, FVF circuit, PVT variation, sensor circuit, Differential Difference Amplifier, operational amplifier

## Abstract

A new power supply rejection (PSR) based enhancer with small and stable dropout voltage is presented in this work. It is implemented using TSMC-40 nm process technology and powered by 1.2 V supply voltage. A number of circuit techniques are proposed in this work. These include the temperature compensation for Level-Shifted Flipped Voltage Follower (LSFVF) and the Complementary-To-Absolute Temperature (CTAT) current reference. The typical output voltage and dropout voltage of the enhancer is 1.1127 V and 87.3 mV, respectively. The Monte-Carlo simulation of this output voltage yields a mean *T.C.* of 29.4 ppm/°C from −20 °C and 80 °C. Besides, the dropout voltage has been verified with good immunity against Process, Temperature and Process (PVT) variation through the worst-case simulation. Consuming only 4.75 μA, the circuit can drive load up to 500 μA to yield additional PSR improvement of 36 dB and 20 dB of PSR at 1 Hz and 1 MHz, respectively for the sensor circuit of interest. This is demonstrated through the application of an enhancer on the instrumentation Differential Difference Amplifier (DDA) for sensing floating bridge sensor signal. The comparative Monte-Carlo simulation results on a respective DDA circuit have revealed that the process sensitivity of output voltage of this work has achieved 14 times reduction in transient metrics with respect to that of the conventional counterpart over the operation temperature range in typical operation condition. Due to simplicity without voltage reference and operational amplifier(s), low power and small consumption of supply voltage headroom, the proposed work is very useful for supply noise sensitive analog or sensor circuit applications.

## 1. Introduction

**Figure 1:**
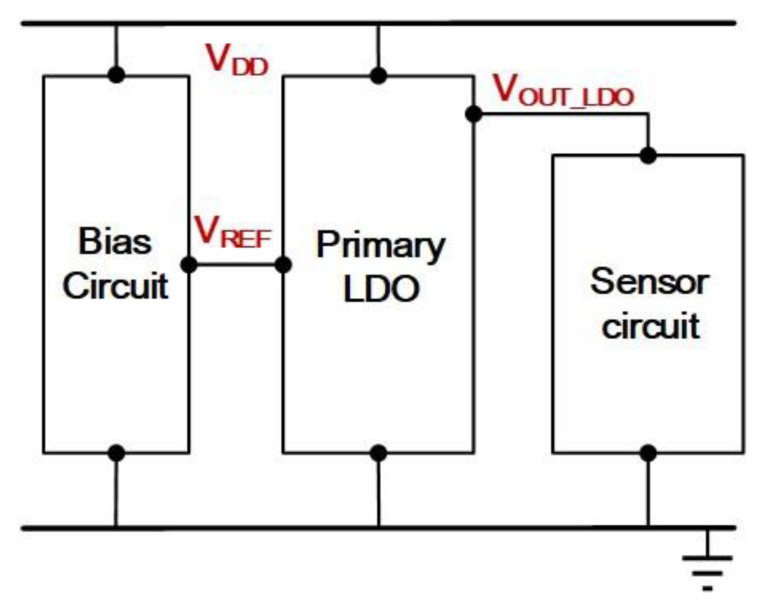
A Sensor Circuit Powered by a LDO Regulator.

With the continuous advancement of integrated circuit design and manufacturing technology, sensor circuits and systems tend to be integrated together on one single chip. For analog circuits, especially sensor circuits, a high-quality supply source is often needed to maintain their function and accuracy. Although specific circuit methods can be employed to improve the performance of sensor circuits arising from the reduced supply sensitivity in VCO based sensor [1] and reduced supply noise in image sensor [2], these methods are only applicable for the limited case examples. In order to increase power supply rejection (PSR), the sensor circuit employing the feedback design [3,4] to the bridge sensing elements is a popular method. However, it relies on splitting a full bridge sensing element into two half bridge sensors. This may not be adequate for many general applications. Due to the feedback mechanism, the loop gain, and the stability become the critical parameters that deal with the PSR performance and frequency compensation, respectively. Besides, other temperature sensors [5,6,7] and an optical mouse sensor [8] have addressed the importance of supply issues based on the impact of supply sensitivity or supply noise on the sensor circuit performance. Regarding recent trend towards low-power consumption, one of the previously reported supply circuits [9] can consume low power. Unfortunately, the circuit topology suffers from the disadvantage of requiring higher supply. Therefore, low-power low-voltage performance becomes one of main agendas in sensor circuit design. The exemplary circuits are low-voltage, low dropout wireless sensor node [10] and low-power low-voltage biomedical sensor IC [11]. Based on the above discussed sensor applications, the Low Dropout (LDO) Regulator is regarded as the common building block used to produce a stable supply voltage to sustain the sensor circuit performance whilst providing adequate PSR as another important characteristic in sensor circuits and systems. Figure 1 shows the general structure of a sensor circuit powered by a LDO regulator in which a voltage reference is generated by a bias circuit to define the output voltage of a regulator.

**Figure 2:**
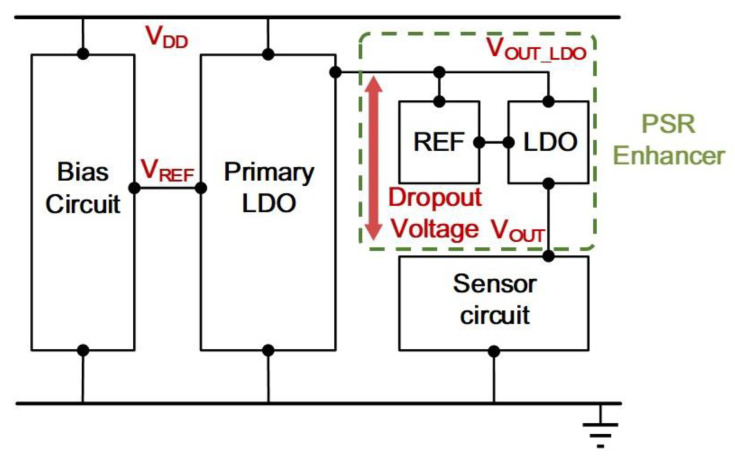
A Sensor Circuit Powered by a LDO Regulator with a PSR Enhancer.

However, in the event that the PSR offered by the regulator is not adequate, a PSR enhancer [12], which usually serves as the secondary regulator, can be employed in analog circuit design. The exemplary circuit block diagram is depicted in Figure 2. As can be seen, the enhancer comprises a voltage reference and a LDO regulator with scaling function. Its output voltage, *V_OUT_*_,_ is designed to be close to the primary LDO regulator output line in which the output voltage is denoted as *V_OUT_LDO_*. The difference between *V_OUT_* and *V_DD_OUT_* of the PSR enhancer defines the dropout voltage. Such the dropout voltage should be made as small as possible in order to offer maximal operation headroom because the price paid for that will be the reduction of sensor supply headroom. More importantly, when the sensor circuit is targeted for low voltage applications, this raises the design challenges about the stability of dropout voltage contributed by the PSR enhancer in context of process, voltage, and temperature (PVT) variation. This is then translated to the problems arising from the stability of the enhancer’s voltage reference, as well as its driving LDO circuit, with the ultimate goal to produce a small dropout voltage which can sustain the proper operation of a PSR enhancer. For an example, consider a dropout voltage of 0.1 V from 1.2 V supply voltage or below in a sensor circuit, this requires a lot of circuit design considerations dedicated to low-power low-supply voltage reference design, as well as scaling regulator design in conjunction with simultaneously addressing PSR concerns. Apart from that, the PSR enhancer serves as an extra block in the sensor applications, thus increasing the cost as the penalty. To relax the issue, the circuit topology of the PSR enhancer should be designed with simplicity. This raises the motivation of this work to design a cost-effective PSR enhancer, not only to improve PSR, but also to produce a stable and small dropout voltage with good immunity against the PVT variation.

## 2. Conventional PSR Enhancer Circuit and Its Design Considerations

PSR enhancers with low dropout voltage are necessary for sensitive or accurate sensor circuits. To meet the requirement of high PSR for sensor circuit in low-voltage low-power applications, circuit design considerations are conducted. At this juncture, MOSFET device operated in the sub-threshold region [13] is often preferred over the Bipolar Junction Transistor (BJT) counterparts [14]. To provide the driving characteristic, a LDO regulator or buffer-like operational amplifier (op-amp) circuit is often needed. Of particular note, the regulator/buffer should be arranged in a separate driving stage design, rather than embedding with the voltage reference in the topology of the merged design. The reason for using cascade topology is that any influence arising from the small dropout voltage will not contribute the stress to the output of the voltage reference stage if it were designed with an embedded buffer. It is important to note that sufficient headroom allowed for the voltage reference output is easily scaled to the targeted dropout voltage through the regulator or op-amp, etc. with a scaling network. Attention is also paid to the PSR issue, which pertains to the circuit topology or frequency compensation technique in regulator or op-amp design. Furthermore, the output stage of the regulator or op-amp buffer should tolerate the change of dropout voltage without deteriorating the open-loop gain function, as the change of dropout voltage will stress the output transistor of the regulator or op-amp based buffer circuit.

**Figure 3:**
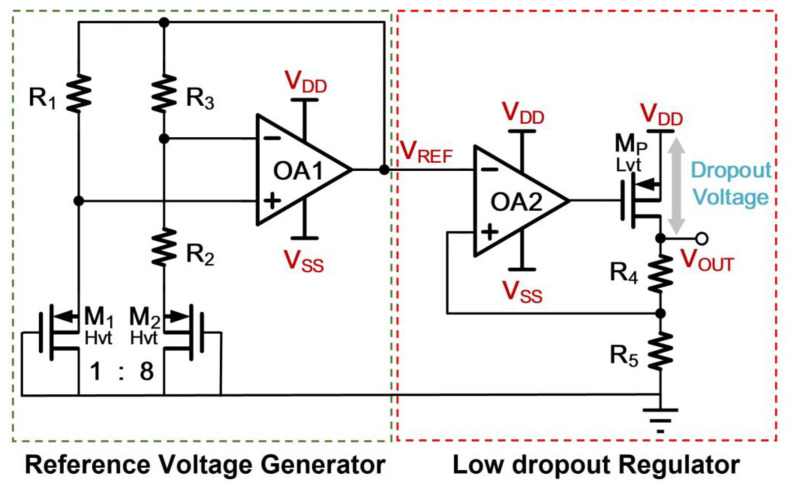
Conventional PSR Enhancer with PSR Design Aware.

Based on these design considerations, an exemplary op-amp based PSR enhancer that features good PSR topology [15,16] for the enhancer design is depicted in Figure 3. The enhancer comprises a reference voltage generator and a LDO regulator.

**Figure 4:**
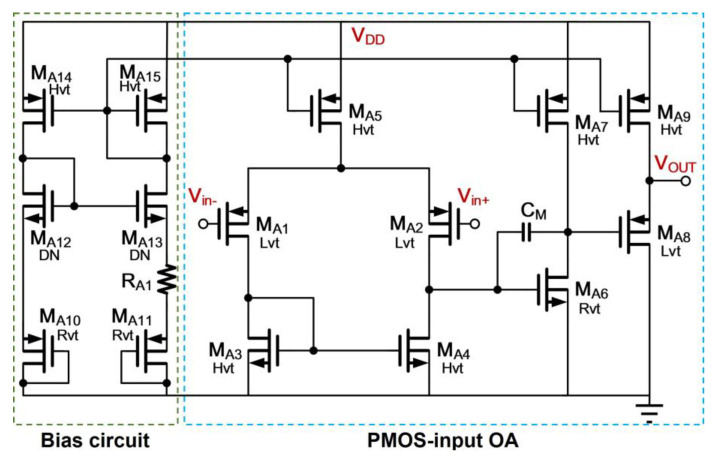
Schematic of *OA*1 for Reference Voltage Generator in Enhancer.

**Figure 5:**
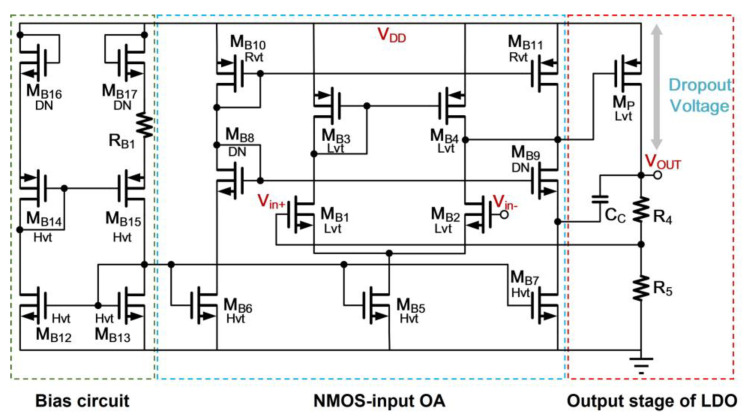
Schematic of *OA*2 for Regulator in Enhancer.

For the Reference Voltage Generator, *M*_1_ and *M*_2_ work in the subthreshold region. As such, the temperature characteristic similar to that of BJT. The *V_REF_*, which is equal to *V_SG_*_1_ plus *V_R_*_1_, is a combination of PTAT and CTAT voltage. The output of *OA*1 yields the reference voltage as:

(1) $$V_{REF}\left(T\right)=V_{SG1}\left(T\right)+\frac{R_{1}}{R_{2}}nV_{T}\ln\left(\frac{S_{2}}{S_{1}}\right)$$

where *S*_1_ and *S*_2_ are the aspect ratio of *M*_1_ and *M*_2_, respectively. Thus, a temperature insensitive, *V_REF_*, can be realized through adjusting the ratio of *R*_1_ and *R*_2_. Besides, the employment of sub-threshold based MOS transistors permits the reference voltage generator to operate at low supply voltage and consume low power. The *OA*1, shown in Figure 4, is a PMOS-input two-stage amplifier with a source follower output stage to avoid the resistive loading effect that influences the open-loop function, resulting in the degradation of PSR. Due to the use of source follower, the output headroom is reduced at the trade-off of *V_REF_* and is unable to produce the small dropout voltage design. Regarding the low-dropout voltage design requirement, *V_REF_* denoted in Figure 3, will be scaled in the LDO regulator through the scale factor (1 + *R*_4_/*R*_5_). To minimize the regulator’s circuit complexity, the power transistor stage can be arranged to cascade with the first-stage differential amplifier *OA*2 to form a two-stage amplifier topology as shown in Figure 5. The well-known cascode compensation technique [17] is applied to obtain a good PSR metric. Finally, the size of each component pertaining to Figure 3, Figure 4 and Figure 5 in the conventional enhancer design is listed in Table 1.

## 3. Proposed PSR Enhancer with PVT-Insensitive Dropout Voltage

### 3.1. Proposed Enhancer Design

**Figure 6:**
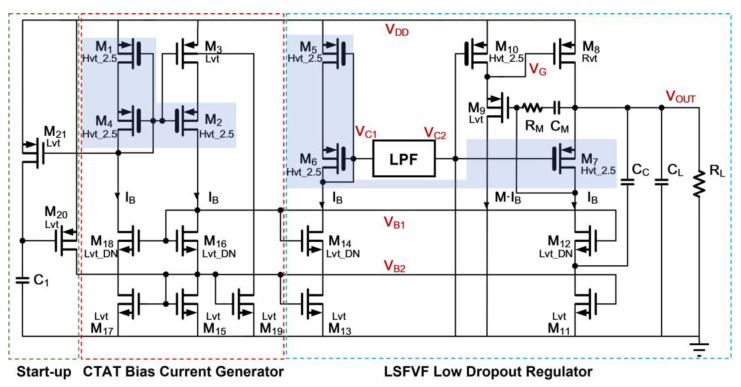
Proposed PSR Enhancer.

The proposed PSR enhancer is shown in Figure 6. It is composed of the Level-Shifted Flipped Voltage Follower (LSFVF) [18] based on the LDO regulator and the CTAT bias current generator with a capacitive start-up circuit. The FVF based regulator has the key advantage of simplicity. The use of LSFVF topology is to relax the power transistor operation headroom at the expense of a slight increase in complexity. This is considered an important design consideration due to small dropout voltage requirement. Moreover, the LSFVF topology also provides a fast response in terms of transient performance, even biased with low quiescent conditions. This helps to reduce the transient spikes in the supply line of low-power sensor circuit. Referring to the LSFVF regulator, it consists of the power transistor *M*_8_, the control transistor *M*_7_, the cascode current source with transistors *M*_11_ and *M*_12_, and the source follower-based level shifter with transistors *M*_9_ and *M*_10_. The intrinsic dc biasing to the control transistor *M*_7_ is obtained from the composite transistor (*M*_5_ and *M*_6_) with the cascode current source (*M*_13_ and *M*_14_) and the pseudo-resistor [19] based low-pass filter (LPF). Further details of the design of LPF will be discussed in the subsequent Section. Of particular note, in order to avoid the influence of leakage effect to the dc biasing of control transistor *M*_7_, high threshold transistors *M*_5_, *M*_6_, and *M*_7_ are employed. Such the biasing implementation has the key advantage of eliminating the complicated voltage generator as well as voltage reference in typical FVF regulator design [20]. Good PSR is still obtained due to the use of LPF for filtering the dc supply noise. As the result, the proposed topology offers a more compact topology with respect to that of the conventional counterpart.

Regarding the biasing circuit in Figure 6, it is designed in a form of a new CTAT current source, comprising transistors *M*_1_–*M*_4_ and *M*_15_–*M*_19_ together with the start-up network comprising transistors *M*_20_–*M*_21_ and a capacitor *C*_1_. This self-biasing network usually performs PTAT current generation in a conventional design. However, by connecting the gate of triode transistor *M*_3_ to the gate of the composite transistor (*M*_1_ and *M*_4_), it is possible that the negative temperature coefficient (*T.C.*) effect of the triode transistor dominates the PTAT effect arising from the current source topology. Consequently, the current source behaves CTAT characteristic. However, the concern is that under high temperature and fast corner case, the transistor *M*_3_ may cut off itself. In order to sustain the operating temperature range for 100 degree C, a limited current is injected to the bulk of *M*_3_ so as to reduce its threshold voltage.

The rationale for this design is that the replica clamping structure formed by *M*_1_, *M*_4_ and *M*_2_ in the biasing circuit matches the clamping structure formed by *M*_5_, *M*_6_ and *M*_7_ in the LSFVF toplogy. Therefore, the design has addressed the tracking issue so as to minimize the impact on the dropout voltage in the presence of process variation. Besides, the generated Δ*V_SG_* in each structure is almost independent of supply voltage change. This is translated to the dropout voltage insensitive to the supply voltage. Finally, referring to the temperature compensation, the generated CTAT current will compensate the change of dropout voltage against the temperature. Consider the output voltage of LSFVF regulator, it decreases with increasing temperature due to the PTAT effect of clamping structure (*M*_5_–*M*_7_). In other words, the increase in dropout voltage comes from the increase in temperature. Besides, it is interesting to observe that if the loop produced by the source-gate volage of *M*_8_, the source-gate voltage of *M*_9_ and the source-drain voltage of M_7_ are made negative *T.C.*, it is able to compensate the positive *T.C.* introduced by the clamping structure (*M*_5_–*M*_7_). However, if a long channel transistor of *M*_7_ is employed with small channel length modulation (CLM) effect, its *V_SD_*_7_ will absorb the temperature-induced voltage change caused by the sum of source-gate voltages through *M*_8_ and *M*_9_. Therefore, it may be difficult to impose the negative *T.C.* voltage change caused by *V_SG_*_8_(*T*) and *V_SG_*_9_ (*T*) on *V_OUT_*(*T*). To tackle the issue, the CTAT current source and the short-channel length *M*_7_ with CLM effect are employed in this proposed design; this permits *V_SD_*_7_(*T*) to behave negative *T.C.* characteristic. Further proof will be given in the subsequent Section. As such, the combined negative *T.C.* contributed by the temperature compensation transistor structure (*M*_7_–*M*_9_) becomes the key part for temperature compensation. In brief, due to the use of the replica structure, simple temperature compensation in the topological network and all transistor-based designs for obtaining a better tracking characteristic, the dropout voltage of the PSR enhancer is almost independent of PVT variation. This yields a stable output voltage from the enhancer to power the sensor circuit of interest.

Regarding the frequency compensation of the regulator, it is stabilized by the cascode compensation [21] in conjunction of Miller RC frequency compensation. This leads to good stability under low quiescent power design.

### 3.2. Low-Pass Filter in PSR Enhancer

**Figure 7:**
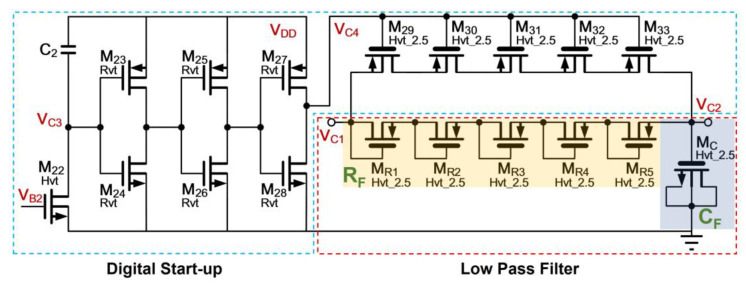
Low pass filter with digital start-up.

The LPF circuit is depicted in Figure 7. Taking into account the small silicon area design, it is based on a first-order filter design using a pseudo resistor *R_F_* and a MOS capacitor C_F_. The pseudo resistor comprises 5 units (*M_R_*_1_–*M_R_*_5_) in series topology to realize a large resistance for use in low frequency, which starts from 1 Hz and above. Due to the extremely large value, high threshold MOS transistors are employed in order to reduce the leakage current. This suggests the potential *V_C_*_1_ is close to *V_C_*_2_. Regarding the MOS capacitor, it is based on a thick-oxide MOS high-threshold transistor with the gate as the top plate terminal and the shorted drain-source and bulk to form the bottom plate terminal. The formation of a large time constant by the LPF will cause the slow start-up of the circuit. To tackle this issue, a digital start-up, which comprises a capacitive start-up network formed by a transistor *M*_22_, six inverter transistors (*M*_23_–*M*_28_), a capacitor *C*_2_ and five MOS switching transistors (*M*_29_–*M*_33_), which are connected in parallel with respective pseudo resistor unit, is proposed. When the system is powered on, a peak voltage of *V_C_*_3_ will appear due to the charging of *C*_2_. Hence, a reversed pulse signal is generated on *V_C_*_4_, which will turn on the switches realized by *M*_29_ to *M*_33_. This allows *V_C_*_1_ to charge *C_F_* rapidly. After the pulse signal, all the switches will be turned off. Then, the LPF establishes a RC circuit with a charged *C_F_* to provide the dc biasing. Of particular note, the off resistance of each MOS switch is not infinite. It will reduce the MOS pseudo resistor unit resistance value when paralleling with a non-ideal OFF switch. This leads to the employment of five serial pseudo resistors. Nevertheless, the effective silicon area of each pseudo resistor is considered small. The penalty for the increase of pseudo resistors is of little concern.

The size of each component, which are pertaining to the proposed PSR enhancer in Figure 6 and the low pass filter in Figure 7 are given in Table 2.

### 3.3. Temperature Analysis of the Building Blocks in PSR Enhancer

#### 3.3.1. CTAT Biasing Current I_B_(T)

When a PMOS transistor works in subthreshold region [22,23,24], the source-drain current *I_SD_*(*T*) is obtained as

$$I_{SD}\left(T\right)=\mu_{p}\left(T\right)C_{OX}V_{T}{}^{2}\left(\frac{W}{L}\right)\cdot e^{\frac{V_{SG}\left(T\right)+V_{tp}\left(T\right)}{nV_{T}}}\cdot \left[1-e^{-\frac{V_{SD\left(T\right)}}{V_{T}}}]\cdot [1-\lambda V_{SD}\left(T\right)\right]\;$$

(2) $$\;\;\;\;\;\;\;\;\;\;\;\;\;=I_{S}\cdot S\cdot e^{\frac{V_{SG}\left(T\right)+V_{tp}\left(T\right)}{nV_{T}}}\cdot \left[1-e^{-\frac{V_{SD}\left(T\right)}{V_{T}}}]\cdot [1-\lambda V_{SD}\left(T\right)\right]$$

where *Is* = *μ_P_ C_OX_V_T_*^2^, *μ_P_* is the carrier mobility, *C_OX_* is the gate oxide capacitance, *n* is the subthreshold slope which is a constant between 1 and 3, *V_T_* = *KT/q* is the thermal voltage, *K* is the Boltzmann constant, *T* is the temperature, *q* is the electronic charge, *S* = *W/L* is the aspect ratio, *W* is the channel width, *L* is the channel length. *λ* is the channel length modulation factor and it has a negative value for PMOS transistor. Further to that, the temperature-dependent threshold voltage and mobility are given as follows:

(3) $$V_{tp}\left(T\right)=V_{tp0}+k_{t}\left(T-T_{0}\right)$$

(4) $$\mu_{p}\left(T\right)=\mu_{p}\left(T_{0}\right)\cdot {\left(T/T_{0}\right)}^{-m}$$

where *V_tp_*_0_ is the threshold voltage at reference temperature *T*_0_ = 300 K, *k_t_* and *m* are constants pertaining to process technology. When *V_SD_*(*T*) > 3*V_T_*, the exponential *V_SD_*(*T*) term in (2) can be ignored and (2) can be rewritten as follows:

(5) $$I_{SD}\left(T\right)\approx I_{S}\cdot S\cdot e^{\frac{V_{SG}\left(T\right)+V_{tp}\left(T\right)}{nV_{T}}}\cdot \left[1-\lambda V_{SD}\left(T\right)\right]$$

Thus, the approximated expression of *V_SG_*(*T*) for a long channel length transistor becomes

(6) $$V_{SG}\left(T\right)\;\approx -V_{tp}\left(T\right)+nV_{T}\ln\left[\frac{I_{SD}\left(T\right)}{S\cdot I_{S}}\right]\approx -V_{tp}\left(T\right)$$

where *I_SD_*(*T*) ≈ *S*∙*I_S_*. To compensate the negative temperature coefficient of the output voltage, a bias circuit without a resistor, which aims to generate an appropriate CTAT bias current, is proposed in Figure 6. *M*_1_, *M*_2_ and *M*_4_ operate in the subthreshold region over the targeted temperature range (−20 to 80 °C). Through selecting same type of high threshold voltage transistor and establishing the replica Δ*V_SG_*(*T*) clamping topology (shaded area) in CTAT current generator with respect to that in core regulator as illustrated in Figure 6, such as the Δ*V_SG_*(*T*) becomes the source-drain voltage across the triode transistor *M*_3_. Therefore, *V_SD_*_3_(*T*) = *V_SG_*_1_(*T*) − *V_SG_*_2_(*T*) = Δ*V_SG_*(*T*). Since *V_SD_*_1_(*T*) and *V_SD_*_2_(*T*) > 3*V_T_*, this gives

$$V_{SD3}\left(T\right)=V_{SG1}\left(T\right)-V_{SG2}\left(T\right)=\left[V_{tp2}\left(T\right)-V_{tp1}\left(T\right)\right]+n\frac{KT}{q}\ln\left(\frac{S_{2}}{S_{1}}\right)$$

(7) $$\;\;\;\;\;\;\;\;\;\;\;\;\;\;\;\;\approx n\frac{KT}{q}\ln\left(\frac{S_{2}}{S_{1}}\right)$$

In view of the identical type of transistor being used for *M*_1_ and *M*_2_, the threshold voltage difference is negligible. Based on (7), *V_SD_*_3_(*T*) can be regarded as a PTAT voltage. *M*_3_ works in linear region to act as an active resistor, the equivalent resistance between the source and drain of *M*_3_ is given as

(8) $$R_{3}\left(T\right)=\frac{1}{\mu_{p}\left(T\right)C_{OX}S_{3}\left[V_{SG1}\left(T\right)+V_{tp3}\left(T\right)-\frac{1}{2}V_{SD3}\left(T\right)\right]}$$

Using (6)–(8), the bias current expression is obtained as follows:

$$I_{B}\left(T\right)=\frac{V_{SD3}\left(T\right)}{R_{3}\left(T\right)}$$

(9) $$\;\;\;\;\;\;\;\;\;\;\;\;\approx \mu_{p}\left(T\right)C_{OX}S_{3}\cdot n\frac{KT}{q}\ln\left(\frac{S_{2}}{S_{1}}\right)\cdot \{V_{tp3}\left(T\right)-\frac{1}{2}\left[V_{tp1}\left(T\right)+V_{tp2}\left(T\right)\right]\}$$

(10) $$\;\;\;\;\;\;\;\;\;\;\;=C_{1}T^{-m}\cdot \left(C_{2}T-C_{3}T^{2}\right)\;$$

(11) $$\frac{dI_{B}\left(T\right)}{dT}=-C_{4}T^{-m}+C_{5}T^{1-m}$$

where

$$C_{1}=\mu_{p}\left(T_{0}\right)T_{0}{}^{m}C_{OX}S_{3}n\frac{K}{q}\ln\left(\frac{S_{2}}{S_{1}}\right),\;C_{2}=V_{tp0\_3}-\frac{V_{tp0\_1}+V_{tp0\_2}}{2}+T_{0}C_{3},$$

$$C_{3}=k_{t1}-k_{t3},\;C_{4}=\left(m-1\right)C_{1}C_{2},\;C_{5}=\left(m-2\right)C_{1}C_{3},$$

Since *M*_1_ and *M*_2_ are the same type of transistors, the *k_t_*_1_ and *k_t_*_2_ are identical. Factor *m* has a typical value of 2.2 for silicon [25], and the parameters *C*_1_, *C*_2_, *C*_3_, *C*_4_, C_5_ are constants with positive value. From (11), it can be deduced that *I_B_*(*T*) exhibits a CTAT characteristic over the temperature range of *T* < *C*_4_/*C*_5_, and the estimation of *I_B_*(*T*) will be discussed in the subsequent Section. Of particular note, the value of *C*_4_/*C*_5_ is above 2*T*_0_ (600 K) which is beyond the operation temperature range of the transistor. Thus, the CTAT bias current *I_B_*(*T*) is used for temperature compensation of *V_OUT_*(*T*). Although *I_B_*(*T*) slightly exhibits nonlinearity, it does not jeopardize the temperature compensation significantly. Due to the fact that the bias circuit is designed with all MOS transistors, it offers better tracking characteristics in terms of process variation as another key advantage. As such, the entire PVT performance will be promising by means of the proposed CTAT current source.

#### 3.3.2. Temperature-Compensated V_OUT_(T) in LSFVF Topology

The output voltage of the enhancer, which is shown in Figure 6, can be expressed as

(12) $$V_{OUT}\left(T\right)=V_{DD}-V_{SG5}\left(T\right)+V_{SG7}\left(T\right)\;$$

Since the transistors *M*_5_–*M*_8_ work in the subthreshold region, it is apparent that *V_OUT_*(*T*) is a CTAT voltage because Δ*V_SG_*(*T*) is a PTAT voltage based on (7).

For *M*_7_, due to the use of a short channel transistor, the CLM is taken into consideration. This yields:

(13) $$V_{SG7}\left(T\right)=nV_{T}\ln\left\{\frac{I_{B}\left(T\right)}{I_{S\;}S_{7}\left[1-\lambda V_{SD7}\left(T\right)\right]}\right\}-V_{tp7}\left(T\right)$$

Substituting (13) into (12), we get

$$V_{OUT}\left(T\right)=V_{DD}-nV_{T}\ln\left(\frac{S_{7}}{S_{5}}\right)-V_{tp7}\left(T\right)+V_{tp5}\left(T\right)-nV_{T}\ln\left[1-\lambda V_{SD7}\left(T\right)\right]$$

(14) $$\;\;\;\;\;\;\;\;\;\;\;\;\;\;\;\;\approx V_{DD}-n\frac{KT}{q}\ln\left(\frac{S_{7}}{S_{5}}\right)+n\lambda \frac{KT}{q}V_{SD7}\left(T\right)$$

From (14), it is obvious that if *V_SD_*_7_(*T*) in the third term of *V_OUT_*(*T*) is made CTAT characteristic, the last two terms will conunteract each other. Regarding Figure 6, the *V_SD_*_7_(*T*) can be written as

$$V_{SD7}\left(T\right)=V_{OUT}\left(T\right)-V_{D7}\left(T\right)$$

(15) $$=V_{DD}-\Delta_{k}-\left[V_{DD}-V_{SG8}\left(T\right)-V_{SG9}\left(T\right)\right]=-\Delta_{k}+V_{SG8}\left(T\right)+V_{SG9}\left(T\right)$$

where Δ*_k_* is the design value of the temperature-insensitive dropout voltage and Δ*_k_* = *V_DD_* − *V_OUT_* = 87.3 mV. Since both *M*_8_ and *M*_9_ work in the subthreshold region, substituting the expressions for *V_SG_*_8_(*T*), *V_SG_*_9_(*T*) using (6), and rewriting (15), we obtain

(16) $$V_{SD7}\left(T\right)\approx -\Delta_{k}-\left[V_{tp0\_8}+V_{tp0\_9}+\left(T-T_{0}\right)\cdot \left(k_{t8}+k_{t9}\right)\right]-n\frac{KT}{q}\ln\left[\frac{I_{S8}\cdot I_{S9}\cdot S_{8}\cdot S_{9}}{M\cdot I_{B}{\left(T\right)}^{2}}\right]$$

where factor *M* is the current mirror ratio between *M*_10_ and *M*_5_. As can be observed from (16), −Δ*_k_* is a constant term, −[*V_tp_*_0_8_ + *V_tp_*_0_9_ + (*T* − *T*_0_)·(*k_t_*_8_ + *k_t_*_9_)] is a CTAT term for PMOS, and the CTAT *I_B_*(*T*) will translate the last term into CTAT counterpart. As a result, *V_SD_*_7_(*T*) yields the CTAT characteristic. Subsituting (16) into (14), *V_OUT_*(*T*) can be rewritten as follows:

$$V_{OUT}\left(T\right)=V_{DD}-n\frac{KT}{q}\ln\left(\frac{S_{7}}{S_{5}}\right)+n\left(-\lambda \right)\frac{KT}{q}\left[\Delta_{k}+V_{tp0\_8}+V_{tp0\_9}-T_{0}\left(k_{t8}+k_{t9}\right)\right]$$

(17) $$\;\;\;\;\;\;\;\;\;\;\;\;\;\;\;\;\;\;\;\;+\left(-\lambda \right)T^{2}\{{\left(\frac{nK}{q}\right)}^{2}\ln\left[\frac{I_{S8}\cdot I_{S9}\cdot S_{8}\cdot S_{9}}{M\cdot I_{B}{\left(T\right)}^{2}}\right]+\frac{nK}{q}\left(k_{t8}+k_{t9}\right)\}$$

(18) $$\;\;\;\;\;\;\;\;\;\;\;\;\;\;\;\;=V_{DD}-N_{1}\cdot T+N_{2}\cdot T+N_{3}\cdot T^{2}\;$$

where

$$N_{1}=n\frac{K}{q}\ln(\frac{S_{7}}{S_{5}})\;N_{2}=-n\lambda \frac{K}{q}\left[\Delta_{k}+V_{tp0\_8}+V_{tp0\_9}-T_{0}\left(k_{t8}+k_{t9}\right)\right]$$

$$N_{3}=-\lambda {\left(\frac{nK}{q}\right)}^{2}\ln\left[\frac{I_{S8}\cdot I_{S9}\cdot S_{8}\cdot S_{9}}{M\cdot I_{B}{\left(T\right)}^{2}}\right]-\lambda \frac{nK}{q}\left(k_{t8}+k_{t9}\right)$$

As indicated in (18), the negated PTAT term -*N*_1_·*T* will be counteracted by the positive CTAT terms which are contributed by dominant linear term *N*_2_·*T* and small quadratic *N*_3_·*T*^2^. They are introduced by the temperature-dependent threshold voltages *V_tp_*_8_(T) and *V_tp_*_9_(T), the design value of dropout voltage Δ*_k_*, the channel length modulation factor *λ* of *M*_7_ and the CTAT current source *I_B_*(*T*). The small quadratic term will display the quadratic effect only at high temperature.

## 4. Results and Discussions

The proposed PVT-insensitive PSR enhancer, as depicted in Figure 6, is simulated using TSMC-40 nm CMOS process technology.

**Figure 8:**
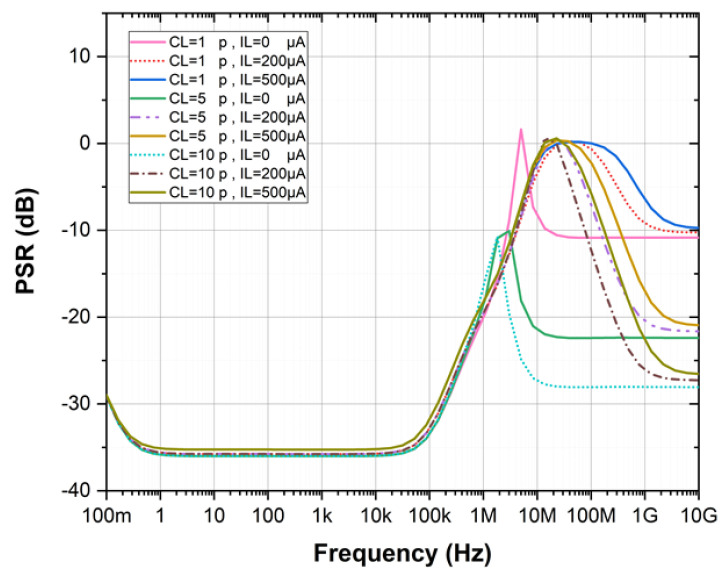
PSR of the Proposed Design at Different Capacitive Loads and Load Currents.

Figure 8 shows the simulated PSR at different capacitive loads and load currents. From Figure 8, the enhancer offers −36 dB PSR from 1 Hz to 100 kHz. In this design, the maximum load current of the enhancer is 500 μA. When the load current exceeds its maximum value, the power transistor *M*_8_ will enter the linear region, and the circuit performance will be compromised. Therefore, there is a trade-off between the driving capacity and the silicon area. In this work, the proposed enhancer is focused on light load current which is less than 1 mA and the typical frequency range for the sensor system is of few MHz or less. Therefore, there is no strict demand on layout issues in view of the insignificant routing parasitics.

**Figure 9:**
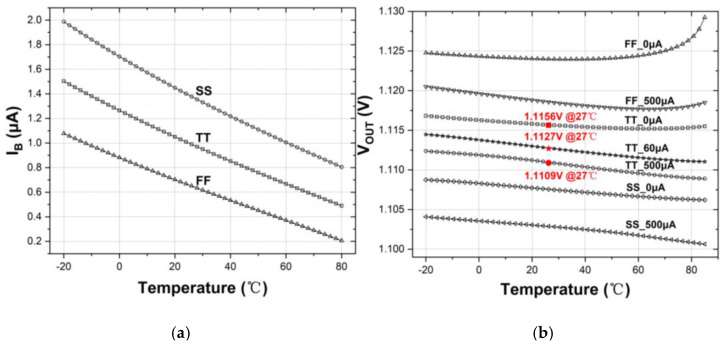
(**a**)Temperature characteristic of *I_B_*(*T*); (**b**)Temperature characteristic of *V_OUT_*(*T*).

Figure 9a illustrates the variation of CTAT bias current *I_B_*(*T*) at different process corners (FF, TT, SS) at the operation temperatures, ranging from −20 °C to 80 °C. The *I_B_*(*T*) decreases with increasing temperatures across the operation temperature range. It shows 0.8 μA under the SS corner at 80 °C, 0.49 μA under the TT corner at 80 °C and 0.2 μA under the FF corner at 80 °C. This confirms the CTAT characteristic as revealed in (10). Regarding the output voltage, *V_OUT_*(*T*), it is evaluated with different process corners, temperatures and loading currents. The simulated results are shown in Figure 9b. Based on the nominal value of *V_OUT_*(*T*) of 1.1127 V at 27 °C in TT case under the load current of 60 μA, the maximum variation is only +1.8 mV/−1.6 mV across two extreme temperature corners. For other load currents of 0 μA and 500 μA, the change of *V_OUT_*(*T*) is +2.9 mV/−1.8 mV at 27 °C. For variation of process corners, *V_OUT_*(*T*) shifts up/down by about +11.3 mV/−9.7 mV from the nominal value case. This is considered acceptably small. Besides, it is observed that *V_OUT_*(*T*) displays an increase at a high temperature of 85 °C under FF corner and little rise at TT corner, this is due to the decrease in *I_B_*(*T*), causing the circuit more sensitive to biasing parameters.

**Figure 10:**
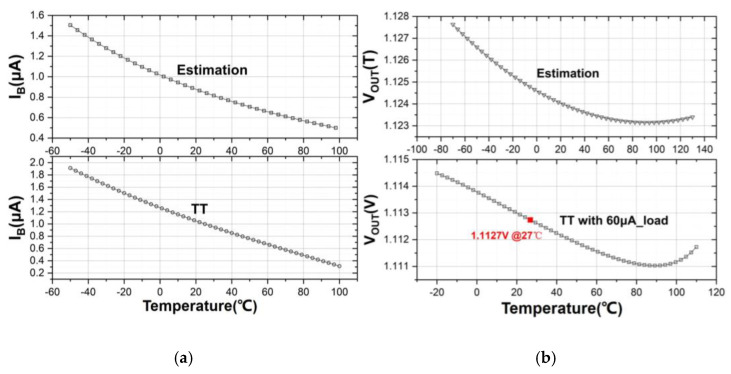
Comparison between theoretical predictions and simulation results under typical case of (**a**) *I_B_*(*T*); (**b**) *V_OUT_*(*T*).

The comparison between the theoretical estimation of *I_B_*(*T*) and *V_OUT_*(*T*) on basis of (10) and (18) and their simulation results are depicted in Figure 10a,b, respectively. It has been suggested that the theoretical predictions correlate very well with the simulation results for both parameters.

**Figure 11:**
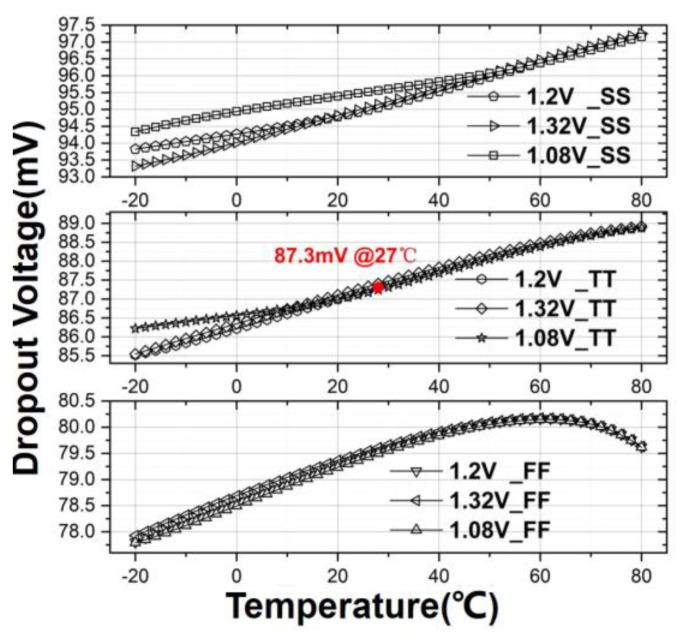
Variation of dropout voltage at different process corners, supply voltages and temperatures.

Besides, different simulations are conducted to observe the dropout voltage under three process corners, 1.2 V ± 10% on *V_DD_* and different operation temperatures in Figure 11. The results have indicated 87.3 mV under the TT corner at 27 °C, 95.2 mV under the SS corner at 27 °C, and 79.5 mV under the FF corner at 27 °C. The dropout voltage has been observed to be almost invariant to the change of supply voltage; a few mV shifts across the entire operation temperature range and about a few mV change over extreme process corners. This led to the total change of +9.9 mV/−9.5 mV under the extreme PVT case consideration. The result has confirmed that the dropout voltage exhibits good immunity against the combined PVT effect.

**Figure 12:**
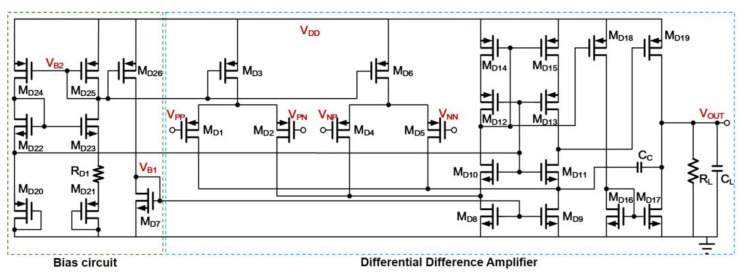
Schematic of the DDA.

To demonstrate the performance of the PSR enhancer for sensor circuit application, a Differential Difference Amplifier (DDA) [26], which serves as the instrumentational amplifier for detecting a full bridge sensor signal is employed. For fair comparison, the conventional enhancer and the proposed enhancer are designed with identical static power consumption and identical supply voltage of 1.2 V using TSMC 40 nm CMOS technology. Table 3 summarizes the static power of building blocks in each design. The schematic of DDA is depicted in Figure 12. It is a standard architecture with the first- being a stage folded-cascade differential amplifier and the second being a non-inverting gain stage with a feedforward path to form the push-pull output stage. The current consumption of the DDA is 60 μA at about 1.1 V supply line from each enhancer. This is treated as the typical operation condition for each enhancer. The performance summary is listed in Table 4.

**Figure 13:**
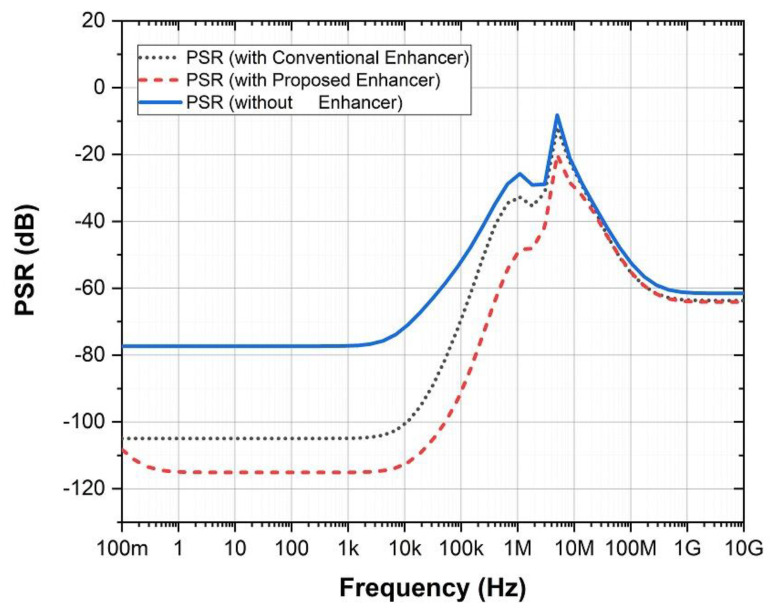
Comparison of PSR for DDA under different design cases.

The comparative simulation results are given in Figure 13 and Table 5. It can be concluded that both low-frequency and high-frequency PSR are improved using the proposed design, with respect to the DDA designs with and without a conventional enhancer.

**Figure 14:**
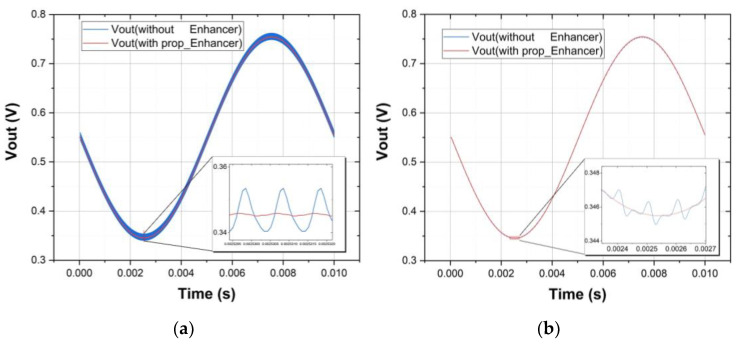

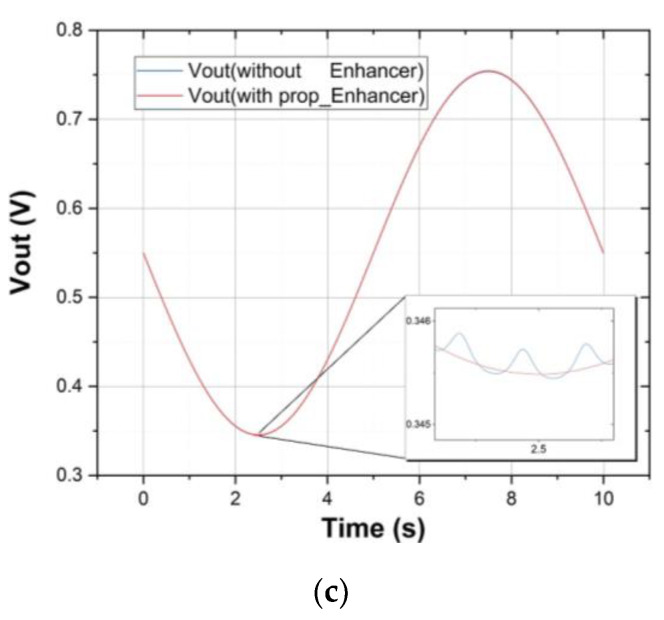
Comparison of time-domain output responses of DDAs with and without proposed enhancer at different noise levels: (**a**) 100 mV_pp_@1 MHz; (**b**) 100 mV_pp_@10 kHz; (**c**) 100 mV_pp_@20 Hz.

For time-domain evaluation, the respective noise signal with amplitude of 100 mV_pp_@1 MHz, 100 mV_pp_@10 kHz and 100 mV_pp_@20 Hz is applied on the *V_DD_* of DDA which is configured with a closed-loop gain of 20. In this simulation, the input common-mode dc signal is 550 mV, whereas the differential-mode signal is 20 mV_pp_. The time-domain output responses of the DDA, are compared with and without the proposed enhancer in Figure 14. It can be observed that the supply noise associated with the amplified input signal is significantly attenuated at the output of DDA.

**Figure 15:**
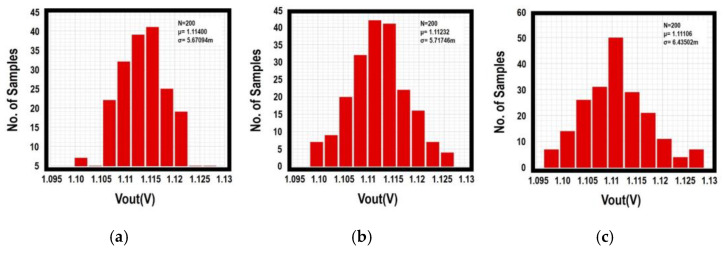

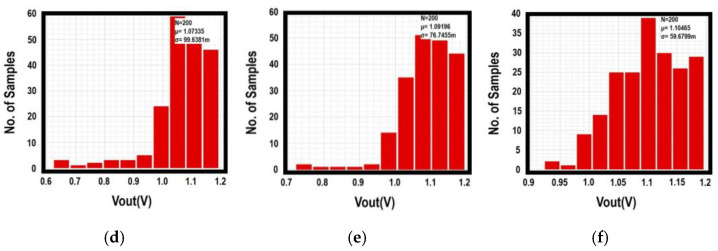
Monte-Carlo simulation of *V_OUT_* of the proposed enhancer (**a**) @−20 °C; (**b**) @27 °C; (**c**) @80 °C; and conventional enhancer (**d**) @−20 °C; (**e**) @27 °C; (**f**) @80 °C.

Figure 15 depicts the spread of output voltage for the proposed PSR enhancer, and the conventional one at a typical load current of 60 μA is compared with the Monte-Carlo simulation runs pertaining to process and temperature variations. With 200 simulation runs, the mean output voltage of the proposed enhancer is between 1.1106 V to 1.1140 V at different temperatures. As observed, the maximum standard derivation is about 6.5 mV across the operation temperature range. On the contrary, the conventional design displays the mean output voltage between 1.0735 V and 1.10465 V, but the maximum standard derivation can reach up to about 100 mV. Compared to the conventional circuit with (i) 2.9% change in mean *V_OUT_* and (ii) Δ*V_OUT_* ≈ 100 mV in maximum standard derivation, the proposed design displays 0.3% change in mean *V_OUT_* and Δ*V_OUT_* ≈ 6.5 mV for maximum standard derivation, respectively. From these results, the proposed design offers very good stability of output voltage in worst case consideration. Consider the process sensitivity, it is defined as (Standard Derivation/Mean value) × 100%. This gives 7.028% for conventional design and 0.514% for the proposed design at 27 °C. This shows that the proposed work has a 14-fold improvement in the reduction of process sensitivity for *V_OUT_*.

**Figure 16:**
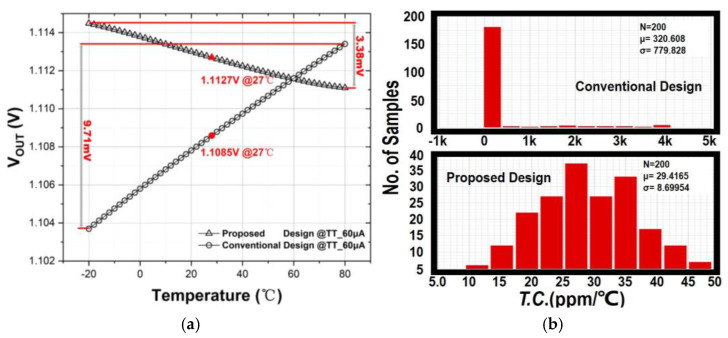
Comparison between conventional design and proposed work of (**a**) temperature characteristic of *V_OUT_* under nominal case; (**b**) Monte-Carlo simulation of *T.C.* of *V_OUT_*.

For *T*.*C*. evaluation, Figure 16a shows the output voltage change against the temperature for the proposed and conventional PSR enhancer under 60 μA typical loading condition and 1.2 V supply voltage, Over the entire operation temperature range, the variation of *V_OUT_* is only 3.38 mV in the proposed design, whereas that of 9.71 mV in the conventional design. This yields the nominal *T.C.* of 30.38 ppm/°C and 87.60 ppm/°C for both circuits, respectively. They are considered comparable in nominal operation conditions. In order to assess the sustainability of *T*.*C*. under process variation, Figure 16b depicts the Monte-Carlo simulation results of the *T.C.* for *V_OUT_* in both circuits. The obtained mean *T.C.* and standard derivation of the proposed work is 29.4 ppm/°C and 8.7 ppm/°C, respectively. These figures are interpreted as at least 10 times and 100 times smaller than those of the conventional counterpart under MC evaluation. It has suggested that it is not easy for the conventional circuit to sustain its output stability against the temperature and process variation when encountering small dropout voltage design.

**Figure 17:**
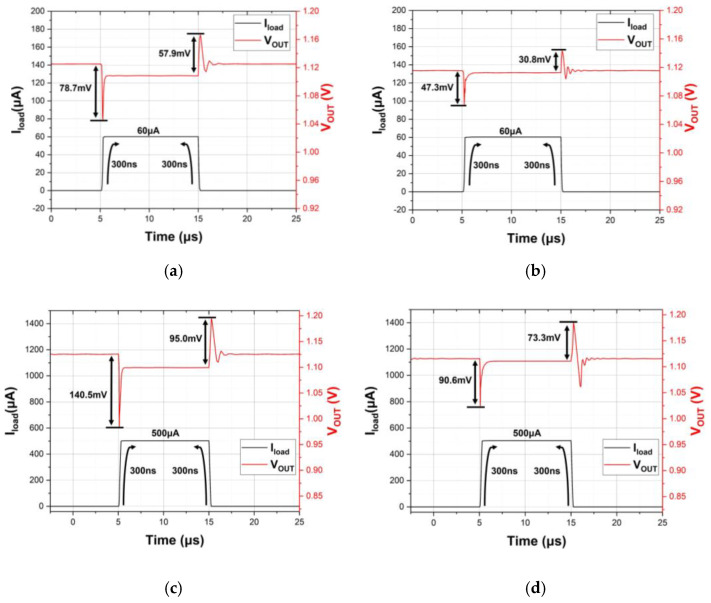
Transient response with 60 μA current pulse of (**a**) conventional design; (**b**) proposed work; and with 500 μA current pulse of (**c**) conventional design; (**d**) proposed work.

Figure 17 illustrates the load transient responses with two load current steps for 60 μA and 500 μA for each enhancer based on the circuit capacitive load of 5 pF. At the edge time of 300 ns, the undershoots of the proposed work are 47.3 mV@60 μA and 90.6 mV@500 μA, whereas the overshoots are 30.8 mV@60 μA and 73.3 mV@500 μA, respectively. Referring to the conventional design, the undershoots are 78.7 mV@60 μA and 140.5 mV@500 μA and the overshoots are 57.9 mV@60 μA and 95.0 mV@500 μA. It can be concluded that the proposed enhancer has achieved smaller undershoot and overshoot. This has demonstrated the advantage of using LSFVF topology for ease of obtaining better transient metrics.

The comparison between the conventional PSR enhancer and the proposed work is summarized in Table 6. As can be revealed, the proposed enhancer offers better performance such as reduced process sensitivity in *V_OUT_*, improved transient metrics, better PSR metrics and simpler circuit topology with respect to those of conventional design at identical power consumption, supply voltage and process technology under low-power circuit design. Further performance enhancement can also be achieved if higher power is allowed in the design.

## 5. Conclusions

A new PVT-insensitive dropout voltage based PSR enhancer on the basis of LSFVF topology dedicated to sensor circuit applications is presented. Its functions are similar to the second regulator, which is inserted between the main regulator and the sensor circuit and is subject to performance degradation under a noisy supply line. Through the proposed topological temperature compensation method, the new CTAT current source, the replica circuit block design approach and all MOS transistors design approaches in critical circuit building blocks for obtaining better tracking characteristics, the proposed work permits a small value of dropout voltage in the design whilst providing good immunity against PVT variation. This is translated to the good stability of output voltage. The circuit is verified by extensive simulation results. Besides, the circuit eliminates the use of operational amplifier(s) as well as the voltage reference. Taking advantage of circuit simplicity, it reduces the silicon area and dissipates low static power consumption. The proposed PSR enhancer and its circuit design techniques will be easily extended to other analog circuit applications, in which the supply voltage headroom, the stability of dropout voltage and the limited circuit’s PSR parameter are of main concern.

## Figures and Tables

**Table 1:** Sizes of the Devices in the Conventional Design.

Device	Size	Device	Size
*M*_1_	40/4 (μm/μm)	*M_B_*_1,2_	10/0.5 (μm/μm)
*M*_2_	320/4 (μm/μm)	*M_B_*_3,4_	10/2 (μm/μm)
*M_P_*	1000/0.16 (μm/μm)	*M_B_*_5_	8.7/1 (μm/μm)
*M_A_*_1,2_	50/1 (μm/μm)	*M_B_*_6,7_	5.6/1 (μm/μm)
*M_A_*_3,4_	3/1 (μm/μm)	*M_B_*_8,9_	2/0.5 (μm/μm)
*M_A_*_5_	2/1 (μm/μm)	*M_B_*_10,11_	10/1 (μm/μm)
*M_A_*_6_	1.7/1 (μm/μm)	*M_B_*_12,13_	5/1 (μm/μm)
*M_A_*_7_	1.4/1 (μm/μm)	*M_B_*_14,15_	2/0.16 (μm/μm)
*M_A_*_8_	20/2 (μm/μm)	*M_B_*_16,17_	4/2 (μm/μm)
*M_A_*_9_	10/1 (μm/μm)	*R*_1_	283 kΩ
*M_A_*_10,11_	6/1 (μm/μm)	*R*_2_	99 kΩ
*M_A_*_12,13_	5/0.2 (μm/μm)	*R*_3_	283 kΩ
*M_A_*_14,15_	1/1 (μm/μm)	*R*_4_	515 kΩ
*R_A_*_1_	18.9 kΩ	*R*_5_	614 kΩ
*R_B_*_1_	27.8 kΩ	*C_M_* for *OA*1	0.8 pF
		*C_C_* for *OA*2	0.4 pF

**Table 2:** Sizes of the devices in the proposed design.

Device	Size	Device	Size
*M*_1_	1.7/0.3 (μm/μm)	*M*_22_	0.32/4 (μm/μm)
*M*_2,4_	20/0.3 (μm/μm)	*M*_23,25,27_	4/0.04 (μm/μm)
*M*_3_	2.1/4.5 (μm/μm)	*M*_24,26,28_	2/0.04 (μm/μm)
*M*_5_	3/0.3 (μm/μm)	*M*_29–33_	0.32/1 (μm/μm)
*M*_6,7_	14/0.3 (μm/μm)	*M_R_*_1-*R*5_	0.32/1 (μm/μm)
*M*_8_	1000/0.16 (μm/μm)	*M_C_*	100/100 (μm/μm)
*M*_9_	10/0.1 (μm/μm)	*R_M_*	5 kΩ
*M*_10_	1.9/0.3 (μm/μm)	*C*_1_	0.1 pF
*M*_11,13,15,17_	4/2 (μm/μm)	*C*_2_	1 pF
*M*_12,14,16,18_	5/1 (μm/μm)	*C_M_*	1 pF
*M*_19_	0.12/5 (μm/μm)	*C_C_*	2.5 pF
*M*_20,21_	1/0.3 (μm/μm)	*C_L_*	1 pF–10 pF

**Table 3:** Power allocation in proposed and conventional enhancer with 1.2 V supply voltage.

Enhancer	Total Power	Bias Circuit	Power Output Stage	Additional Block
Proposed Work	4.75 μA	1.96 μA	0.98 μA	None
Conventional Design	4.75 μA	1.96 μA	0.98 μA	*V_REF_* Generator 8.67 μA

**Table 4:** Performance summary of the DDA.

Supply Voltage	Power Consumption	*R_L_*	*C_L_*
1.1 V	60 μA	100 kΩ	30 pF
**Open Loop Gain**	**PSR**	**CMRR**	**Bandwidth**
77.8 dB	−77.4 dB@1 Hz	87.4 dB	315 Hz
**Unit Gain Frequency**	**Phase Margin**	**Input-Referred Noise**	
2.2 MHz	69°	$97\;\mathrm{nV}/\sqrt{\mathrm{Hz}}$@1 kHz	

**Table 5:** Comparison of PSR at low and high frequency for DDA under different design cases powered about 1.1 V from the respective enhancer with 1.2 V supply.

Frequency	Without Enhancer	Conventional Enhancer	Proposed Enhancer
1 Hz	−77 dB	−105 dB	−115 dB
1 MHz	−26 dB	−32 dB	−50 dB

**Table 6:** Performance comparison of the simulation results between the conventional PSR enhancer and the Proposed Work at Typical Case.

	Conventional Design	This Work
Process Technology	40 nm CMOS	40 nm CMOS
Power Transistor Size	PMOS (1 mm/160 nm)	PMOS (1 mm/160 nm)
Current Consumption *I_Q_* (μA)	4.75	4.75
Supply Voltage (V)	1.2	1.2
*V_OUT_* @60 μA_Load (V)	1.1085	1.1127
Minimum *I_LOAD, min_* (μA)	0	0
Maximum *I_LOAD, max_* (μA)	500	500
Δ*I_LOAD_* (μA)	500	500
Voltage Reference Required	Yes	No
Op-amp Required	Yes	No
Δ*V_OUT_* (−20–80 °C) (mV)	9.71 ^1^	3.38 ^1^
PSR Bandwidth (kHz)	65.5	107.2
PSR @ 1 Hz, 1 MHz (dB) Mean of *V_OUT_* (V), 200 samples	−31.6, −8.1 1.0920	−36.0, −20.2 1.1123
SD of *V_OUT_* (mV), 200 samples	76.7	5.72
*T.C.* (1 sample @nominal) (ppm/°C)	87.60 ^1^	30.38 ^1^
Mean *T.C.* (200 samples) (ppm/°C) SD *T*.*C*. (200 samples) (ppm/°C)	320.6 ^2^ 779.8	29.4 ^2^ 8.7
Process Sensitivity for *V_OUT_*	7.028%	0.514%
Edge Time (μs) Δ*V_OUT_* (mV) @500 μ*A*	0.3 140.5	0.3 90.6
Edge Time Ratio *K*	1	1
FOM^3^ [27] (mV)	1.33475	0.86070

^1^ At TT corner with 60 μA load condition ^2^ Monte-Carlo simulation results under 60 μA load condition and *T.C.* = [Δ*V_OUT_*/(Δ*T* × *V_OUT_normal_*)] × 10^6^ ppm/°C, *V_OUT_normal_* = *V_OUT_* @27 °C ^3^ FOM = *K·*Δ*V_OUT_* *· (I_Q_ + I_LOAD, min_)/ΔI_LOAD_.*

## Data Availability

Not applicable.
